# Creating a sense of place when implementing a new emergency department in Denmark: a qualitative study

**DOI:** 10.1186/s12913-024-11980-3

**Published:** 2024-11-27

**Authors:** Jeanette Wassar Kirk, Per Nilsen, Ove Andersen, Nina Thórný Stefánsdóttir, Mette Bendtz Lindstrøm, Byron J. Powell, Tine Tjørnhøj-Thomsen

**Affiliations:** 1 Department of Clinical Research, Copenhagen University Hospital, Amager and Hvidovre, Hvidovre, Denmark; 2 Department of Health and Social Context, National Institute of Public Health, University of Southern Denmark, Copenhagen, Denmark; 3 Department of Health, Medical and Caring Sciences, Linköping University, Linköping, Sweden; 4 Emergency Department, Copenhagen University Hospital Hvidovre, Hvidovre, 2650 Denmark; 5 Department of Clinical Medicine, University of Copenhagen, Copenhagen, 2200 Denmark; 6 Center for Mental Health Services Research, Brown School, Washington University in St. Louis, St. Louis, MO USA; 7 Center for Dissemination & Implementation, Institute for Public Health, Washington University in St. Louis, St. Louis, MO USA; 8 Division of Infectious Diseases, John T. Milliken Department of Medicine, School of Medicine, Washington University in St. Louis, St. Louis, MO USA

## Abstract

**Background:**

Physical locations play an essential yet often overlooked role in healthcare implementation processes. Implementation Science frameworks such as the Theoretical Domains Framework, the Consolidated Framework for Implementation Research, and the Implementation in Context framework acknowledge the importance of the physical environment, but they often treat it as a passive backdrop for change. However, from a cultural geographic perspective, spaces and places are dynamic, influencing behavior, social structures, and the acceptance of new practices. This study aims to explore how managers and emloyees develop a sense of place in a new emergency department (ED) and how these spatial dynamics influence the implementation process.

**Methods:**

This study used a multi-sited ethnographic design, tracking the implementation process across multiple hospital locations from 2019 to 2023. Fieldwork was conducted in settings such as management meetings, micro-simulation training, and tours of the new ED construction site. A total of 53 participants, including managers, nurses, and physicians from 12 specialized departments, were purposively selected. Data were collected through ethnographic field notes (750 single-spaced pages) and semi-structured interviews averaging 39 min. Analysis was guided by situational analysis and cultural geography, integrating human and nonhuman elements. An overall inductive approach was used to develop theory from observations through analysis, applying a coding system to identify key themes related to spaces, places, traces, and sense of place.

**Results:**

Five themes emerged regarding the development of a sense of place: (1) comfort, influenced by physical elements such as daylight and indoor climate; (2) spatial organization, affecting collaboration, workflow, and professional identity; (3) familiarity, highlighting cultural practices and equipment in fostering belonging; (4) time, where construction delays enabled deeper emotional engagement; and (5) involvement, showing that initial criticism transformed into stronger ownership through increased engagement.

**Conclusion:**

This study highlights the importance of a sense of place during pre-implementation of new physical locations in healthcare. Factors such as comfort, spatial organization, familiarity, time, and involvement are key to participants’ development of a strong sense of place in the new ED. These insights are crucial for designing implementation processes that address both physical and emotional needs, influencing outcomes such as acceptability, adoption, and sustainability.

## Background

In healthcare, implementing new programs, interventions, or organizational changes often involves physical locations. Implementation science (IS) frameworks, such as the Theoretical Domains Framework (TDF), the Consolidated Framework for Implementation Research (CFIR), and the Implementation in Context (ICON) framework, recognize physical locations under various domains: environmental context, inner setting, and organizational climate and structures, respectively [1–4]. These frameworks suggest that physical locations can influence implementation outcomes, yet there is often a lack of detailed analysis on how and why these locations matter within the IS literature [5].

Concepts such as context, setting, and environment are frequently used interchangeably in IS [6]. Generally, when physical settings are mentioned in the IS literature, they are conceptualized as a stable, passive backdrop for the implementation of various changes [1, 3]. This perspective assumes that the location of implementation is inconsequential to the process [5]. However, we might need to distinguish between the implementation of changes in practices (what people do) and changes in physical locations (where people do something). IS mostly deals with practice changes, which makes it logical that aspects such as physical locations are not usually in focus [7].

Ignoring the role of physical locations in people’s interactions and practices may result in important elements in the implementation process being missed. When physical locations are part of these processes, is it merely about participants adapting over time to the new environment? From a cultural geographic perspective, a familiar space is seen as a unique composition of physical, social, and symbolic traces left by people or activities [8]. Changing physical locations and relocating, such as implementing a new work routine that requires the use of a different hospital ward, can disrupt an employee’s sense of identity and belonging, affecting their acceptability of the implementation process [5]. Studies emphasize the importance of personalizing workspaces to help employees maintain or re-establish their identity and comfort in new physical locations [9].

This article highlights the potential importance of considering physical locations as dynamic and influential elements in the pre-implementation process phase. This is an unique perspective as the existing literature from e.g. evidence-based design research typically examines sense of place post-occupancy (10,11). We argue that physical locations are not merely a static and neutral frozen entity or a passive backdrop for human interactions and practices; they should be understood and examined as dynamic and active elements [8]. If physical locations actively influence implementation processes, they can have an impact on outcomes, including the acceptability and adoption of the implementation object. Here, we look to cultural geography for insights into how human cultures and their practices are connected to the physical and social spaces they inhabit. This involves the study of how people create, adapt, and transform their physical surroundings, as well as how these surroundings influence their behavior, identity, and social structures [8, 10].

A cultural geographic perspective distinguishes between space, place, and environmental context, referring to the conditions influencing a location. Space and place involve the physical and social dimensions of areas and their perception [11]. The pronoun “their” refers to areas, emphasizing the focus on how these areas are perceived both physically and socially by people. These concepts often overlap and understanding them can enhance IS by recognizing how geographic distribution, spatial disparities, and physical locations affect implementation success. Integrating these factors into IS helps tailor implementation strategies to specific dynamics, improving their real-world effectiveness [8, 10, 11].

Our previous studies on implementing a new emergency department (ED) in a Danish university hospital highlighted the role of physical locations in participants’ workflows, interprofessional collaborations, and material processes, such as transferring equipment and adapting to the layout [12, 13]. The new ED also had an emotional impact on participants, posing additional challenges for management. This raises questions such as: How did the physical space in the new ED affect social interactions and hierarchies between different professional groups? In what ways were the new physical environments in the ED shaped by or adapted to the existing cultural norms and practices within the hospital? These observations prompted the development of the present study, which aims to explore the role of spaces and places in the development of a sense of place among managers and employees in a new ED, and how this sense of place, along with the traces left, influenced the pre-implementation process. The concept of sense of place and traces is further described in the theoretical section.

## Theoretical foundation

### Cultural geography

Cultural geography can provide insights into how physical locations influence implementation processes and outcomes by examining the relationship between humans and their environments [7–9, 11]. This field distinguishes between “space” and “place” [8].

Space generally refers to the physical or abstract dimensions of an area without regard for its cultural or social significance. It is a neutral, geometrically defined concept associated with numerical aspects such as distance, size, and location, and lacks specific cultural or historical context [8].

In contrast, place extends beyond mere physical location to encompass the meaning and significance attributed to a space [10]. Place integrates cultural, social, and historical values, as well as personal experiences and memories. It involves the emotional, social, and symbolic attachments that individuals or communities develop with a specific area. Places are not static; they are dynamic constructs central to human existence, shaping and being shaped by our lives. Understanding and managing both space and place is crucial for comprehending and potentially transforming the social world.

Cultural geography examines the impact of spaces and places on various levels: micro, meso, and meta. Scholars have explored how environments affect mental health [14], the relationship between gender and urban spaces, and how this influences the exclusion of certain groups from these urban spaces [15, 16], and how urban spaces shape the identity of cities, particularly in political contexts and social conflicts [17, 18].

Implementation processes inherently involve transformation at individual, collective, or organizational levels [19]. These processes are influenced by practices and contextual factors, including management styles and professional identities [12], and hence it could be argued that spaces and places could be significant to implementation processes. The participants involved in or affected by the relocation may perceive it as successful or not depending on their sense of place. Thus, spaces and places may have a significant impact on implementation outcomes. For instance, in healthcare, professionals’ identities and practices are shaped by the spaces where they interact and procedures are performed, thereby endowing these spaces with significance and meaning [12].

### Sense of place

The concept of “sense of place” [20, 21] explores the material and immaterial elements that define the relationship between individuals and specific locations [10]. Sense of place refers to the unique attributes of a location, encompassing both positive and negative aspects [11]. Over time, individuals engaging with a place develop a sense of place, and those with a profound connection to a specific place are said to have a stronger sense of place [8]. Places lacking individuals’ sense of place are referred to as being “placeless,” indicating a lack of personal or organizational attachment. Sense of place integrates emotional, experiential, and affective traces that tie individuals to physical locations and shape their identity, suggesting that “who we are is fundamentally connected to where we are.”

### Traces

According to Anderson [8], a place’s identity is shaped by the “tangle of traces” created by the daily activities of its users. These traces can include anything from physical objects, such as a well-worn path or a piece of furniture, to intangible elements such as memories, traditions, and social interactions [10]. Over time, these traces embed history and meaning into a place, influencing how people perceive and interact with it. These traces are not neutral; they represent values, ideas, or attitudes of specific groups, influencing a place’s identity. Consequently, traces determine who can experience a sense of belonging to a place. The power of traces is evident in how they regulate activities, lifestyles, and languages in specific locations, affecting how places are used and experienced. This connection underscores the influential role of traces in shaping a place’s sense of place [10, 21].

### Implementation of a new ED in Denmark

In recent years, Danish emergency services have undergone significant reforms driven by the Danish Health Authority [22, 23]. These reforms aim to centralize emergency services to enhance access to specialized facilities and reduce the risk of inappropriate admissions [23]. New EDs are being constructed based on three political visions: patient-centered care with single rooms for privacy, increased effectiveness through reorganization and technology, and hospitals that are flexible and adaptable to future needs [2]. Hospitals in western societies symbolize advanced medical care and technological progress, contributing to strong attachments to this organizational form [24].

This study focuses on a new ED under construction at a university hospital in the Capital Region of Denmark, set to open by autumn 2024. The new ED will feature a centralized entry, an increase in the number of patient beds from 29 to 92 for stays of up to 48 h, and continuous presence of specialist physicians [24]. The redesign will separate the emergency and medical units by floors; the emergency unit will be on the ground floor and the medical unit on the first floor. The current ED mixes medical patients regardless of specialty; the new ED will categorize the first floor into specialty clusters, such as cardiac or infectious disease units (for further descriptions, see Kirk et al. [13]). This significant change in location, organization, practices, and relationships may have an impact on implementation outcomes.

### The new ED from a cultural geographic perspective

Seen through the concepts of sense of place and traces, the new ED is both a cultural phenomenon and a social creation influenced by healthcare professionals, managers, patients, and relatives. Places are not static but shaped by social, cultural, and political processes, therefore their meanings and identities can be negotiated and reinterpreted based on the users and the prevailing power dynamics. A cultural geography approach is highly relevant for understanding the implementation of the new ED because it can help explore the meaning and identity attributed to the new ED and the physical locations by its users. It can also contribute to an understanding of the role of spaces and places in the development of a sense of place among managers and employees in the new ED, and how this sense of place, along with the traces left, influences the pre-implementation process. As participants develop their sense of place, they will negotiate and leave material and immaterial traces that influence the significance and use of the new ED during the pre-implementation phase, determining how the spaces should be used and who should navigate or inhabit them.

## Design and methods

The implementation of a new ED is inherently dynamic, marked by extensive organizational changes over more than 4 years and involving a complex, multi-layered process affecting numerous individuals and groups. This dynamic nature is characterized by continuous shifts, adaptations, and developments throughout the implementation process, necessitating an analytical approach capable of capturing these evolving aspects. Consequently, the new ED (the implementation object) is defined by its evolving implementation process rather than a fixed, static location.

This study uses a multi-sited ethnographic design, which involves following the continuous implementation process across spaces and places [25]. Multi-sited ethnography is distinguished not only by fieldwork conducted in multiple geographic settings but also by following the connections and movements between these sites [26]. The fieldwork led us to various locations, from the meeting room in the ED to the Board of Directors corridor, to micro-simulation training [27], to the unfinished concrete floors of the new building under construction (new ED). The focus of this article is exploring the development of a sense of place among participants, how it evolves from a space to a place, and its significance for the implementation process and outcomes.

### Participants

Participants were purposively selected to include those actively involved in the implementation process. This group comprised chief managers, middle-level managers, and key employees such as nurses and physicians with implementation responsibilities, representing 12 specialized departments (Table 1). All these specialized departments will be integrated into the new ED.

**Table 1:** Participating departments

Specialty	Department
Medical specialty	Department of Cardiology
	Department of Gastroenterology (medical)
	Department of Infectious Diseases
	Department of Internal Medicine (including Department of Respiratory Medicine and Department of Endocrinology)
Surgical specialty	Department of Orthopedic Surgery
	Department of Gastroenterology (surgical)
Emergency specialty	Emergency Department
Other	Department of Clinical Biochemistry
	Department of Obstetrics and Gynecology
	Department of Pediatrics and Adolescence Medicine
	Department of Radiology

### Data collection

Data were collected primarily through an ethnographic field study and recorded in field notes from 2019 to 2023, documenting weekly management meetings and tours of the construction site. These meetings included the existing ED management team (12 participants), a nurse responsible for education, and two consultants from the current ED. Over the 4-year period, additional participants relevant to the implementation were included in meetings and tours according to our sampling strategy. We created an open observation matrix with three columns: (1) observations; (2) reflections; and (3) analytical remarks or memos, applied during all field activities, including meetings and micro-simulation training [24]. After each session, we updated the notes with additional reflections and analytical insights [27]. The author group engaged in continuous discussions, sharing reflections, and extracting key points from the field notes. A total of 750 single-spaced pages of field notes were taken in relation to the management meetings. Part of this material was included for analysis in this study, which is described in the analysis section.

In addition, oilcloth sessions (micro-simulation training) were conducted to train staff and managers on new patient pathways and the new building design [13, 27]. After these sessions, all 53 participants were interviewed in hospital meeting rooms or their offices. The duration of the interviews averaged 39 min (ranging from 26 min to 1.03 h). The interview guide was developed and pilot-tested by NTS and JWK and covered eight topics. One topic was developed for this study, focusing on the participants’ experiences with the physical locations and their sense of belonging to the new ED (Table 2). The topic focusing on the physical location covered 90 pages and was also included for analysis in this study. Data collection was carried out by NTS and JWK.

**Table 2:** Examples of interview questions

Belonging to the new emergency department (sense of place)	Introduction: We are interested in knowing if, as a healthcare professional, you feel a sense of belonging to the places and buildings where you work (and therefore, to understand if this is something the interviewee can recognize)
	You know that you will be working in the new ED in 2025. To what extent do you feel a sense of belonging to the new ED?
	What does it take for you to establish a sense of place to the new ED and the physical buildings already?
Possible additional question	Do your physical work environments contribute to defining who you are professionally?

### Ethical considerations

The study was approved by the Danish Data Protection Agency (ID no. VD-2019-160). The Research Ethics Committee of the Capital Region of Denmark deemed formal ethical approval unnecessary for this non-biomedical study, so formal ethical approval was not obtained. All participants provided both oral and written informed consent, adhering to the principles of the Helsinki Declaration [28]. We applied situational ethics, integrating organizational context with a sense of responsibility, morality, and intuition throughout the interviews [29]. For instance, we explicitly communicated the conditions of anonymity and emphasized our independence from the hospital’s Board of Directors.

JWK, a researcher with a nursing background and experience in qualitative research, was familiar with the organization and the emergency medical specialty. NTS, an anthropologist with experience in qualitative research, initially had limited knowledge of the organization and emergency medicine but became acquainted with these aspects throughout the study.

## Analysis

The analysis is informed by situational analysis (SA) [30], which complements cultural geography by addressing the complexity of cultural geographic phenomena [31]. SA focuses on the situation under study, encompassing both human and nonhuman elements and their interactions [32]. Key aspects of SA that guide this study include (1) the importance of materialities within the situation and (2) the necessity for researchers to create memos reflecting on their experiences, positions, and reflections.

In this study, memos derived from the comprehensive ethnographic and interview data and were used to identify and select relevant theoretical concepts. The identification process involved JWK systematically reviewing these memos, which served as reflections on specific situations and field observations, where materialities, spaces, places, sense of place, and traces played central roles. For example, a memo discussing the impact of physical space on participant interactions highlighted the theme of “Familiarity within the new ED,” directly linking it to our claim regarding the significance of place in cultural geographic phenomena. By analyzing and comparing the content of these memos, the researchers were able to identify patterns and themes that pointed to specific theoretical concepts relevant to the focus of the study. Thus, the memos functioned as an analytical tool, helping the researchers select and ground their theoretical framework in empirical observations.

A detailed coding system was developed based on the chosen theoretical concepts. This system included specific codes such as " materialities,” “traces,” and “sense of place,” each representing key themes identified in the memos. Data were then systematically reviewed and analyzed according to this coding system, leading to the identification of themes and empirical examples (see Table 3). This systematic approach ensured a thorough exploration of the data and reinforced the connection between the identified themes and the theoretical concepts.

**Table 3:** Examples of themes and sub-themes

Themes	Sub-themes	Content	Quotes
Comfort of the new emergency department (ED)	Environmental factors (daylight, sound, indoor climate)	Participants highlighted the importance of natural light, sound conditions, and indoor climate in creating a positive sense of place	“Many participants agreed that daylight, sound conditions, indoor climate, and more space than their current physical facilities would create a positive relationship to the physical location because these factors would constitute a foundation for a good physical working environment.” (Interview no. 17)
	Increased space	The provision of more space in the new ED was viewed as a significant improvement. Having more room is expected to enhance workflow, reduce overcrowding, and contribute to a more comfortable and efficient work environment	“Well, you could say that there are so many people in our office now that it’s causing problems … it’s very hot in there, and you can’t open the windows. I don’t know how it will be; I don’t even know where the offices will be located. If it’s still like this, where you can’t open the windows, I mean. There are so many students, including medical students, so we’re practically sitting on top of each other, right? And you have to do it because you need to sit down and document things, even though you could use those mobile stations, but… you just don’t.” (Interview no. 13)
Spatial organization in the new ED	Impact on workflow and collaboration	Participants expressed concerns about how the physical arrangement of spaces affects daily workflows and interdisciplinary collaboration. Proximity among specialties is seen as crucial for efficient communication and patient care, with the spatial organization directly influencing the effectiveness of professional interactions	“The large office (12 people) should have visibility over all 72 beds from this office. This again relates to workflows because I believe this is where the evening and night shifts will be stationed, and they will want to have an overview.” (Management meeting, January 2023)
	Sense of community and professional identity	Spatial organization plays a key role in fostering a sense of community and professional identity. Shared spaces, where nurses and physicians work closely together, enhance mutual support and cohesion, creating a positive work environment. Conversely, separating these groups disrupts this sense of community and challenges their professional identities, leading to frustration	“Ask the staff what is most important to them. Involvement is a tool for achieving this.” (Management meeting, June 2022)
	Challenges to specialty identity and belonging	The relocation of certain units, such as moving the medical unit to a different floor, raised concerns about changes to specialty identity. The new spatial organization risks altering how staff perceive their roles, with potential negative impacts on their sense of belonging and resistance to the implementation of the new ED	“Those who work in an emergency medical setting are challenged because when specialists take the complex and perhaps interesting patients, it becomes very professionally unchallenging.” (Interview no. 6)
Familiarity with the new ED	Cultural practices and specialty identity	Familiar physical layouts and specific equipment, such as reclining chairs or examination rooms, symbolize the cultural identity of specialties, reinforcing a sense of belonging and defining each group’s unique practices within the ED	“Forgotten, you say? Yes. For example, in meeting series or other instances. And we have many times pointed out that while we may not be part of the medical unit, and we’re not part of the gastro unit, which has to give up a lot of beds and so on, we are part of the medical area [fumbles for words], and we have a relatively large share of the patients that come into the new ED, and it could… And it might not be planned for a significant change for our specialty, but we might still have some input.” (Interview no. 24)
	Emotional responses to spatial changes	When familiar layouts or equipment are altered or shared among specialties, it can trigger strong negative emotions and resistance, because these changes challenge established cultural practices and the sense of place for healthcare professionals	“Yes, I think it does. Because this idea of being on all the time—24/7? Yes, and you might have to do some different tasks than you’re used to, and it’s also a bit hard to put into words because we actually don’t know for sure yet. It’s something I think you’ll figure out once you get out there in the new ED.” (Interview no. 42)
	Traces and spatial control	Special equipment serves as traces that shape the identity of spaces, with the placement of such equipment indicating which specialty controls and belongs in a particular area. These traces play a significant role in fostering or hindering a sense of place, influencing how professionals engage with the new ED	“So, it’s a huge concern about the spaces all the way down. And it also means that you can see that some … (thinking pause) changes are happening in the composition of the staff. Some staff are choosing different paths. This is partly because the physical premises will not be as they expect.” (Interview no. 3)
Time to construct the new ED	Delays as both an obstacle and an opportunity	Delays in constructing the new ED are seen as economically problematic, negatively affecting participants’ perceptions. However, some view the delays as beneficial, providing extra time to build relationships, understand the space, and foster a deeper connection with the evolving sense of place	“Could you write about the road that is causing a lot of issues for the staff – the ambulance road. The road that was supposed to take 5 weeks has now taken 4 months.” And “We know that more time is needed at the start – it is an investment.” (Management meeting, March 2022)
	Impact on sense of place	The delay in construction allows participants to form stronger emotional and social connections with the new ED, fostering a robust sense of place and ownership. In contrast, a rushed transition could hinder these connections, resulting in a weaker sense of place and reduced positive impact on the implementation process	“I don’t think they have understood how significant an organizational change this is. I’m not sure they realized it’s a very large organizational change. It will need to be addressed again.” (Management meeting, February 2021)
	Time-related instability and its consequences	Disruption of daily routines, such as changes in meeting times, can create confusion and instability, which are essential to maintaining a stable work environment. This instability may lead to resistance and a weakened connection to the new ED, potentially undermining the project’s success	“LO says that they ‘have slept on the job here’ by not installing nitrous oxide. SK adds that a standalone nitrous oxide system costs SEK300,000.” (Management meeting, February 2023)
Involvement in developing the new ED	Initial resistance and sense of distance	Healthcare professionals initially felt excluded from decision-making, leading to criticism, a sense of detachment, and decreased engagement with the new ED	“Yes, I think it’s something we need to be told about, like what it actually means for us and for our functions, our jobs, and the department” and “Well, we need to collaborate, or as mentioned, we will have both gastroenterology, gynecology, and I think maybe even the pediatric department as well, right? And I know that many of my colleagues say they hope they won’t have to be in, let’s say, the gastroenterology specialty or track or whatever it will be called, because we still don’t know how it will affect our professional expertise. So, we would prefer to avoid that.” (Interview no. 9)
	Efforts to foster ownership and engagement	Managers worked to involve specialists and shift attitudes by increasing their participation in planning and decision-making, which gradually fostered a sense of ownership and connection to the new ED	“I think people need to be involved so that they feel like a part of it … That they feel they are part of it … That they are heard. That their thoughts about what could be beneficial are listened to, right?” (Interview no. 22)
	Time as a catalyst for attitude change	Through experiences and strategic involvement, attitudes transformed from negativity to support, with healthcare professionals developing a stronger sense of place and acceptance of the new ED	“The idea behind the Wednesday meetings is to establish working groups that will make progress. We have already seen how attitudes can shift over time at these meetings. We are getting familiar with the new ED.” (Management meeting, August 2019)

## Results

Five key themes emerged regarding the creation of a sense of place during the pre-implementation phase. The first theme, comfort of the new ED, encompasses physical aspects, such as daylight, sound, indoor climate, and space organization, which collectively influence participants’ experiences. The second theme, spatial organization within the new ED, involves the impact of layout on collaboration, workflow, professional identity, and specialty affiliation. The third theme, familiarity with the new ED, highlights the role of cultural practices, and layout and equipment enhance a sense of belonging and familiarity with physical spaces. The fourth theme, time to construct the new ED, addresses the effects of construction delays, which provide opportunities for deeper engagement, emotional connections, and adaptation to changes in work schedules. The final theme, involvement in developing the new ED, reflects on initial criticism and distance due to lack of involvement at the early stage, but over time, greater engagement fostered ownership and a stronger sense of belonging.

These findings connect to related cultural geographic concepts such as sense of belonging and sense of community, offering a deeper understanding of how geographic factors influence the creation of a sense of place and affect the pre-implementation process.

### Comfort of the new ED

The analysis showed that most of the participants discussed the physical comfort of the new facilities at the ED. Especially, after the have taken a tour during the construction of the new ED. Many participants perceived that daylight, sound conditions, indoor climate, and more space than their current physical facilities would create a positive sense of place because these factors would constitute a foundation for a good physical working environment. Dialogues that focused on how the physical spaces in the new ED were organized analytically, referred to as spatial organization, were more prevalent in the data material.

### Spatial organization within the new ED

Spatial organization refers to how places are arranged within a specific geographic area [10]. Participants expressed strong concerns about the physical arrangement of spaces on the 1st floor and how the spaces would affect their workflow and collaboration. In a local manager meeting, an emergency medicine physician expressed:

It does matter how we are physically organized around the specialties in the new ED. For example, we often collaborate with gastroenterologists when a patient comes in with stomach pain, so it would be beneficial for efficient collaboration and communication if we are not physically distant from each other [in the four clusters] instead of being placed a bit away from everything. (Meeting, April 2019)

Participants highlighted the importance of knowing the physical location of healthcare professionals because proximity affects daily workflows, interprofessional collaboration, and routines. There was a particular emphasis on the need for easy accessibility to foster coordination and communication, not just for physicians but also for nurses and patients. A chief nurse pointed out with an assertive tone of voice:

It is important for the nurses to be able to find the right physicians to attend to the patients…and important for the patients to know which cluster they are placed in, and which physicians they can expect to attend to them. It provides security for our nurses and patients which reduces stress … it will have an impact on their support for the implementation of the new ED. (Meeting, June 2019)

Here, security for nurses refers to relational security: knowing where to find physicians and specialists, which reduces uncertainty and stress, thus enhancing efficiency and patient care. Relational security allows nurses to feel a sense of control in their work, and patients benefit from knowing what to expect and who will care for them. This underscores the critical role of spatial organization in creating a sense of place. Nurses expressed the need to know their colleagues’ locations and specialties to foster close interdisciplinary collaboration. Effective spatial organization supports convenient communication, improving workflow and creating a sense of security for both nurses and patients. Conversely, being “a bit away from everything” was seen as detrimental to collaboration and communication. Furthermore, healthcare professionals’ perceptions of the four clusters were shaped by the physical layout and the social and emotional interactions within these spaces, contributing to a sense of security and trust in professional relationships.

In several interviews, participants emphasized the vital role of spatial organization in developing a sense of place within the new ED. Surgical specialists, in particular, voiced concerns about the inadequate size of meeting rooms and the potential issue of separating physicians and nurses into different rooms. A physician from the surgical specialty explained:

In our current department, physicians and nurses are placed in the same room. This means that we all hear the same overview of the patients, we easily keep track of which nurse is responsible for which patients, and socially it has an impact, where we often eat together during the evening shift, etc. Well, it contributes to creating a sense of community and a good working environment. (Interview no. 11, 2021)

Bringing nurses and physicians together in the same space fostered a strong sense of community, defined by mutual support and cohesion. This phenomenon can be characterized as the perception of a collective of individuals sharing common interests, values, or goals. In such an environment, there is a palpable presence of mutual support and cohesion among the group members [5]. This sense of community, facilitated by spatial organization, was crucial for shared understanding and collaboration in patient care, as well as for creating a positive social work environment. This sense of community transformed the space into a place. Another surgeon continued expressing an ongoing concern:

In the new ED, the rooms are much smaller. There doesn’t seem to be an opportunity for physicians and nurses to be placed together. It is concerning because it is a part of our specialty identity that we are placed together and do things together. It is a continued frustration. (Interview no. 14, 2021)

The separation of physicians and nurses had far-reaching implications, affecting not only practical workflows but also social and professional identities. Spatial positioning became a key factor in defining each specialty’s identity. The importance extended beyond their placement in the same room, affecting how the space influences and reinforces each specialty’s unique identity. The lack of shared spaces and communal activities disrupted the sense of community, leading to frustration and emotional challenges during the implementation process.

Changes in the physical layout, as noted in interviews and management meetings, included separation of a ground floor (focusing on injuries) for patients believed to have an in-hospital stay of less than 6 h and a 1st floor (focusing on medical diseases) where patients could be admitted for up to 48 h, as opposed to the current 24 h. A middle manager from the ED explained her concerns:

Today, it is easy for the staff to collaborate because the two units are located on the same floor. However, now the medical unit is moving to the 1st floor. How can we ensure that the collaboration continues to function? Furthermore, we have an image of the bed ward being located upstairs in the hospital, not our ED. So, if our medical unit is associated with a bed ward and a greater focus on basic nursing, that is not desirable because our emergency nurses do not want to be identified with that. As managers, we face a significant task in changing this perception. (Meeting, May 2022)

This change in spatial identity (moving to the 1st floor perceived as a ward) raised concerns because nurses from the acute specialty might not identify with a ward. Moving the ED nurses challenged both the nurses’ spatial and professional identity, understood as changes that questioned or affected the nurses’ perception of and relationship with the physical location where they work. A bed ward was associated with other collective characteristics and traces, such as a greater emphasis on basic nursing, than the emergency nurses collectively wanted to be identified with. This possible shift in professional tasks had a negative influence on their specialty identity and their sense of belonging in the new ED. The risk associated with the new identity generated resistance to the implementation of the new ED:

It was not what we had envisioned with the implementation of the new ED. (ED nurse, Interview no. 7, 2023)

Spatial organization became an important factor for developing a sense of belonging and connection to one’s specialty, as well as being a part of something bigger. The physical separation of different specialties and professionals posed challenges in establishing a positive sense of place in the new ED, having a negative impact on communication, collaboration, and workflow, which ultimately hindered the implementation process.

### Familiarity within the new ED

Participants highlighted activities or equipment related to specific places in connection with the establishment of the new ED. A physician from a surgical specialty recounted:

We have discussed extensively with the managers of the existing ED the importance of having a separate place for our acute patients. We now have that in our current department. It works quite well for us [referring to the surgical specialty]. What is also characteristic is that in the room, we have reclining chairs for the patients, not beds. That is also unique. Having such a place would be important for us, if we are to achieve a sense of belonging in the new ED. It is important the places are familiar to us. (Interview 23, 2021)

Data showed how familiarity with physical layouts and their usage over time created cultural practices that became meaningful for participants within a specialty, also fostering a sense of belonging to a particular group. These practices reflected the underlying values, norms, and social structures unique to each specialty. This sense of belonging was expressed through specific spatial organizations for particular activities, such as the admissions of emergency patients, where material elements such as reclining chairs played a role. Unlike the standard use of beds in most EDs, reclining chairs served as a cultural trace, symbolizing the surgical specialty’s identity, and demarcating their space. This process imbued the place with a distinct identity for the healthcare professionals who used it.

If the familiar physical layout did not become an option, the risk of health professionals perceiving physical spaces as meaningless increased, regardless of the barriers to building a positive or negative sense of place. When familiar physical layouts and organizations were challenged as part of implementing a new ED, it generated clear resistance that revealed the influence of cultural practices and habits in the formation of the participants’ sense of place.

In terms of interior design, the new ED was configured with fewer examination rooms than the participants desired. Not all decisions about locations and physical layout were finalized when the management meetings or oilcloth sessions were held. During discussions about the layout of, for example, examination rooms, the current ED managers suggested that the rooms could be flexible, allowing multiple specialties to use the spaces. This elicited emotional reactions, as conveyed by physicians from the cardiology specialty:

I don’t know what they’re thinking. We can’t have gynecological examination beds set up in a place where we’re supposed to examine patients with heart diseases. Such beds do not belong to our specialty. It sends shivers down my spine that they can’t understand how crucial it is for us [staff within a medical specialty] that the rooms are furnished with our equipment. It matters for our experience of the new ED. (interview no.33, 2023)

Traces in the form of special equipment become significant for shaping these rooms and fostering a sense of belonging among healthcare professionals from different specialties. When rooms were furnished with equipment from another specialty, intended to create flexibility, it often triggered strong negative emotions and a sense of detachment. This increased the likelihood of perceiving the place as “placeless,” turning it into a mere space. For instance, gynecologic examination beds became more than just neutral equipment; they served as regulatory traces, indicating which specialties could access specific rooms and how. Thus, discussions about equipment were imbued with a power element that extended far beyond the tangible equipment but contributed to defining who had control over specific rooms in the new ED. This dynamic ultimately strengthened the sense of placeness for healthcare professionals from certain specialties in the new ED.

The analysis thus showed that familiarity with places shapes how the participants perceive and engage with the environments. The more familiar or well-known a place is, the stronger the sense of belonging, which enhances the participants’ sense of place. Traces, in the form of physical marks, such as examination rooms or beds, and emotional experiences, leave imprints that shape the identity of the place. These traces can be inclusive or exclusive, determining who can occupy or inhabit the space, thus influencing the participants’ sense of place and positively supporting the implementation process.

### Time to construct the new ED

Situations and discussions among participants that centered on time, particularly the duration of the construction phase of the ED, indicated that time played a crucial role in shaping their sense of place. Delays in the construction of the ED were considered inconvenient for many reasons, including in economic terms. However, some participants believed that these delays were an advantage, as one middle manager nurse explained:

It is, of course, a bit annoying that the construction is being delayed, but the upside is that it gives us time to get to know each other and the physical locations better. I think that’s an advantage so that we don’t continue to have a sense of distance when discussing the new ED. (Interview 51, 2022)

From a cultural geography perspective, the delay in building construction was not seen as an obstacle, but as an opportunity for a deeper engagement with the evolving sense of place. The time spent in anticipation and tours allowed participants to form more nuanced and personal connections with the new ED, even before physically occupying it. This extended period enabled them to engage in dialogues with colleagues and managers, thereby influencing their understanding and attachment to the future ED.

We suggest that the process of imagining and discussing the new ED is viewed as part of the active creation of its identity and significance. Rather than simply being speculative, these interactions contribute to shaping how the physical locations will be perceived and experienced once it is operational. By involving participants in these pre-occupancy discussions, the organization fosters a sense of ownership and belonging that might not emerge as strongly with a more immediate transition.

Furthermore, from a cultural geographic perspective, the emotional and social connections formed during this period were seen as vital in shaping how the space will ultimately be inhabited and used (being a place) in the future. The delay allows for a gradual and thoughtful integration of the new ED into the participants’ professional and emotional landscapes, resulting in a more robust and enduring sense of place.

In contrast, a rushed physical occupation into an unknown space might hinder participants’ ability to develop these connections and adapt, leading to a weaker sense of place and reduced positive impact on the implementation process. Thus, the delay should be considered a strategic advantage in cultivating a meaningful and well-integrated sense of place.

Conversely, for some participants, the delay of the physical occupation meant increased distance from the new ED. A medical secretary expressed:

I can sit in my current office and watch the building rise up… but when the move-in date keeps getting postponed, I can’t relate to the new ED. It’s a bit paradoxical that I am physically close but feel distanced. (Interview 19, 2020)

Even though several participants were physically close to the construction building of the new ED, which could imply an experience of a closer connection to the new ED and thus the creation of a sense of place, the postponement of the physical occupation meant the opposite. Several participants felt less connected, resulting in a “sense of placeless.”

Another temporal aspect discussed involved changes in physical schedules in the short term, for example, when times at the management meetings were altered. As a physician reflected:

It might not seem like a big deal, but when meetings are moved from 12:00 PM to 9:00 AM in the morning, it’s a mess. It creates confusion and instability in the daily work routines. Stability that is crucial for a positive work environment. We end up giving up on the idea of the new ED. (Meetings, September 2023)

Time changes for meetings affected the daily routines and structures of places. This aspect of the sense of place is crucial for participants to maintain a stable and predictable work situation. Stability and predictability in work situations are, as illustrated above, fundamental for creating a healthy and efficient work environment. In contrast, instability seemed to increase potential resistance against the idea of the new ED.

### Involvement in developing the new ED

From the beginning of the implementation process in 2019, many of the specialists, in both surgical and medical specialties, were very critical of the political decision to establish a new ED. A physician expressed his dissatisfaction:

There are many aspects, including the physical layout, that have been completely incomprehensible to us. From the start, the Board of Directors has given us the experience of being involved in decisions. Especially in the first couple of years, we felt a significant distance from the new ED. (Meeting, January 2023)

Participants described a sense of distance from the new ED as a result of insufficient involvement and influence in decision-making regarding the physical layout. The consequence of participants feeling a sense of distance to the ED was that their engagement and investment in the implementation of the new ED declined. Throughout the 4-year period, the managers of the current ED worked to influence the participants’ experience of being involved and to create ownership for the new ED. One manager described:

It has been hard work to change the attitude of the specialists regarding feeling ownership and a sense of belonging to the new ED. (Meeting, May 2023)

The effort to change attitudes was noticed by several of the specialists. A physician from the gastroenterology specialty observed how attitudes changed over time:

Time has been a factor for us. At the beginning of the process [implementation], we were very negative. Today, our attitude is different. The new ED has also become ours; the physical places are arranged to suit our specialty, and it is clear that we have created a stronger connection with the new ED. Now we support the idea of the new ED. (Meeting, November 2023)

The analysis showed that time became an important factor in shaping health professionals’ sense of place, both positively and negatively. Time allowed experiences, memories, and historical events to influence the way the health professionals perceived and connected to the new ED. Delays in making the new ED fully operational provided managers with an opportunity to implement change management strategies, involving healthcare professionals in planning and decision-making regarding the physical layout and workflows. This increased ownership and engagement, transforming neutral spaces into meaningful places and strengthening their sense of place.

The factors identified in the results pertain to various aspects of the pre-implementation process. Comfort and spatial organization are perceived based on how participants envision their experience in the new ED and their understanding of the layout. These factors relate to the design process, while familiarity and involvement are more linked to organizational change management, and the time factor pertains to the construction process. Collectively, these factors may influence the development of participants’ sense of place from diverse approaches, and cultivating this positive sense of place during pre-implementation can enhance acceptance and adoption of the new ED.

## Discussion

This study aimed to explore the role of spaces and places for the development of a sense of place among managers and employees in the new ED and how this sense of place, along with the traces left, affected the pre-implementation process. Five key themes were identified as central to participants’ experiences regarding how spaces and places influenced managers’ and employees’ sense of place in the new ED, and how this, along with the traces left, affected the pre-implementation process: the comfort of the new ED, spatial organization, familiarity with the new environment, construction timelines, and involvement in the development process.

### Spatial organization and its impact on collaboration and implementation

Spatial organization, or how physical spaces are arranged, was one of the emerging themes. The arrangement of spaces, such as clustering relevant specialties together, was seen as essential for effective collaboration and communication. The spatial organization of the new ED was pivotal in cultivating a sense of belonging among participants. Perceived proximity to one another helped foster a connection to their specialties and the broader organizational mission. This underscores the idea that the physical environment has a profound impact on social dynamics and professional identity, both of which can affect the acceptability of the relocation and other implementation outcomes [6, 12]. However, the physical separation of specialties posed challenges.

Research by Coyle [33] highlights how planned and unplanned interactions, or “collisions,” within shared spaces can facilitate idea exchange and collaboration. These interactions are best supported by open layouts that encourage meetings and interactions. In the 1970s, Allen [34] demonstrated that communication declines sharply as workspace distance increases, with a marked decrease in communication beyond 8 m. The new ED’s two-floor design risks creating specialty silos [35], potentially limiting informal interactions and collaboration crucial for both the implementation and functioning of the ED. Thus, characteristics of spatial organization were perceived to be essential for fostering a sense (positive or negative) of belonging and connection among participants.

Despite the literature emphasizing that physical separation between specialties in healthcare settings can offer several benefits, such as improved efficiency, privacy, and infection control [36–38], it may hinder the development of a cohesive environment and have a negative impact on communication, collaboration, and workflow. This spatial challenge could impede the adoption and integration of the new ED. Effective spatial design is therefore essential for successful implementation; Rogers’ theory highlights the importance of broader environmental factors in adoption [39]. Effective spatial design could optimize these factors and support successful implementation.

### Familiarity and sense of place in implementation processes

Familiarity with the new ED’s physical layout was significant for participants. Elements reflecting existing environments, such as specific equipment and room layouts, enhanced a sense of belonging and professional identity. This was evident from the traces, that is, the emotional or physical marks left by participants that shape their connection to a place [8]. For example, participants negotiated a preference for reclining chairs over beds in surgical areas, which underscores how specific features can reinforce a specialty’s identity and sense of place. Familiarity with physical locations can affect how participants perceive and interact with their environments [8, 10]. Thus, designing implementation processes to align with users’ familiarity can be expected to influence their acceptance and effectiveness [37]. This finding aligns with Kreitman’s research [40] which highlights the impact of emotional and historical significance in fostering inclusivity and support.

The CFIR emphasizes considering physical space attributes and their effects on users [3], and the Basel Approach for Contextual Analysis (BANANA) supports evaluating historical and emotional factors to tailor implementation strategies [41]. By integrating these physical, historical, and emotional elements, implementers can design spaces that align with users’ sense of place, thus improving acceptance and the success of the implementation. Massey [14] described that to translate a “sense of place” into concrete design, the focus should be on local culture, the use of materials and forms that reflect the location, and the creation of spaces that foster an emotional connection to the surroundings.

Resistance to changes in spatial organization and equipment underscores the importance of retaining familiar elements to ensure the successful implementation of the new ED; research on workplace design indicates that maintaining established practices can ease transitions and mitigate adverse effects on professional identity [42]. In addition, involving participants in the decision-making process during pre-implementation can reduce resistance significantly [43, 44]. The theory of organizational readiness for change [45] emphasizes that user involvement enhances the perceived value of change, increasing support and facilitating smoother adaptation. When participants feel valued and included, they are more likely to accept changes in physical locations, contributing to a positive implementation process [46]. Therefore, careful planning and execution of spatial changes, considering employees’ professional identities and established practices, are essential for successful implementation.

### Inclusivity and exclusive spaces

Power dynamics are intricately woven into the physical and emotional traces that define a place’s identity. Elements such as examination beds, and the emotions they evoke, can either foster inclusion or create exclusion among participants, significantly affecting their sense of belonging and influencing who feels comfortable occupying them. Power is thus exercised through the control and design of physical spaces, influencing who feels connected to or alienated from the environment [8]. Integrating both physical and emotional aspects into the design of spaces can enhance inclusivity and effectiveness, leading to broader acceptance of the new ED [43]. Our results underscore this by showing that resistance to changes in spatial organization often stems from discomfort with new layouts and equipment. Maintaining familiar elements and addressing emotional responses, as our findings suggest, can mitigate resistance and align with the notion that power dynamics in space design affect participants’ inclusion or exclusion. Therefore, understanding how power structures affect experiences and the sense of belonging is crucial for fostering engagement and ownership. Addressing these dynamics while creating positive emotional connections can improve the overall implementation process [44]. Thus, effectively navigating power dynamics in space design and implementation is essential for a successful, inclusive, and equitable process [45].

### The dual role of time in shaping the sense of place

Time played a dual role in shaping participants’ sense of place. Construction delays provided an opportunity for deeper engagement with the new ED, particularly from a management perspective. The extended timeline allowed for more extensive planning and dialogue, helping to build a sense of ownership and attachment to the new space [10]. Initiatives such as oilcloth sessions supported this ownership by fostering a sense of control and involvement among stakeholders [13].

Conversely, these delays also introduced a sense of distance and detachment, especially among employees who were physically close to the new ED but felt emotionally disconnected [47]. This paradox illustrates the complexity of time as a factor in the sense of place. Extended planning periods can enhance a sense of belonging but may also intensify feelings of distance if not managed carefully. The results showed how emotional disconnect and feelings of distance are deeply rooted in the spatial and social dynamics of the workspace [47]. To mitigate these effects and leverage a positive sense of place, we suggest that implementation strategies should include active staff involvement in the design process, create opportunities for interaction and feedback, and foster a supportive community within the new space. These approaches can enhance the sense of place and contribute to more successful implementation [48, 49].

Our findings suggest that implementation outcomes are closely linked to physical, organizational, and temporal factors. Effective spatial organization, familiarity with the physical locations and artifacts, active user involvement, and proper time management can all influence outcomes such as acceptability, adoption, and sustainability [50].

Compared with the findings of Proctor et al. [51], our analysis underscores the critical importance of certain factors in shaping implementation outcomes. Our current study highlights these factors, demonstrating how their careful consideration can significantly influence the effectiveness of implementation efforts. By building on and extending the insights from the previous review, we aim to address these gaps and offer a more nuanced understanding of how these critical factors contribute to successful implementation outcomes.

The relevance of the sense of place theory varies depending on the context. When changes are made to the physical setting, this theory provides valuable insights into the significance of place. However, if changes pertain to practices within an unchanged setting, the theory may be less relevant. Its applicability should be empirically examined in each context to determine its significance.

### Future research

**Fig. 1:**
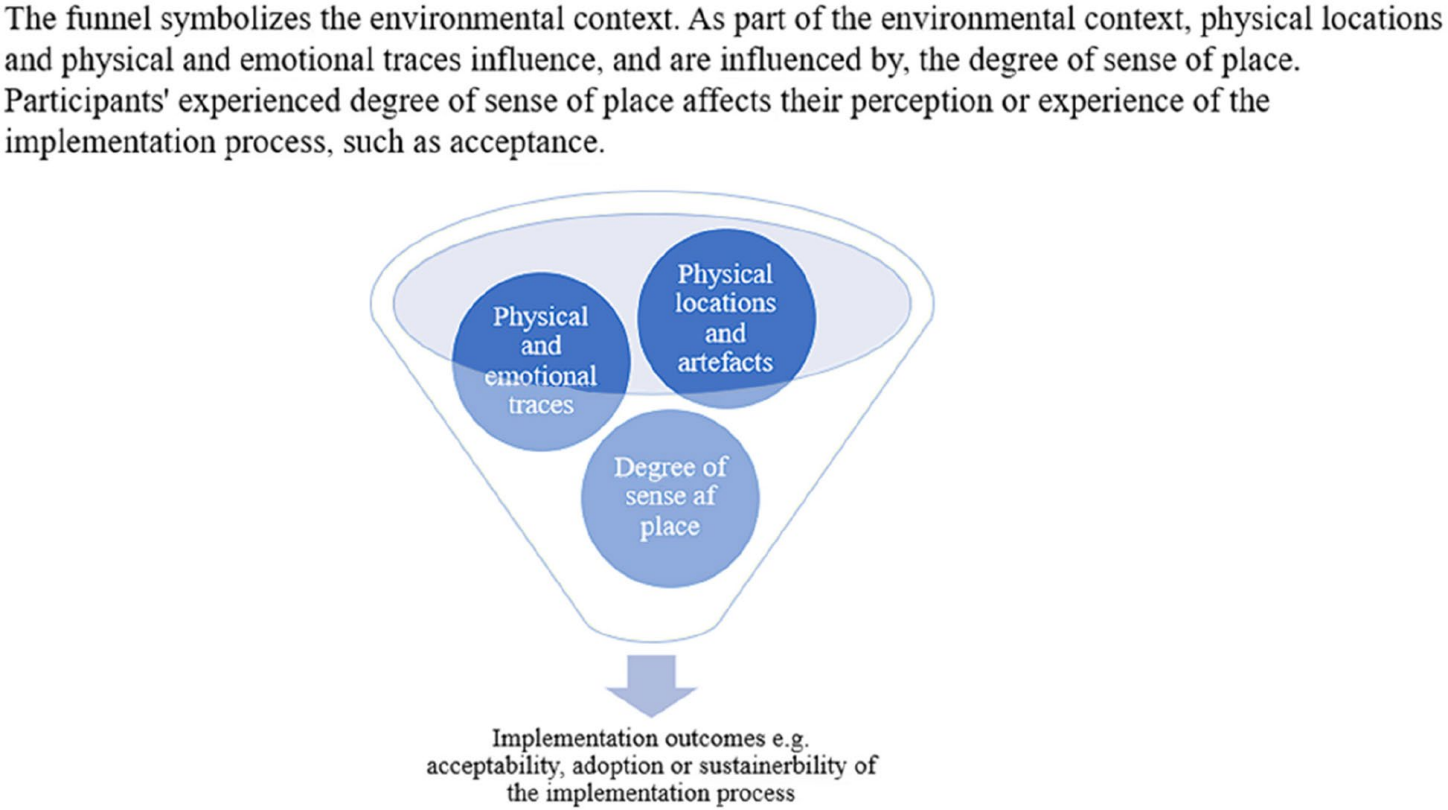
Cultural geographic factors influencing implementation outcomes

The findings of this study can be summarized in a theoretical model that shows factors influencing implementation outcomes. Developing conceptual models that integrate the sense of place with implementation theory could provide important insights for managing implementation processes in various organizational environments. Thus, we propose the model shown in Fig. 1.

Environmental context serves as the overarching concept or background influencing all other elements in the model. Within this context, physical locations and artifacts are key components that directly shape and are shaped by the other elements. The result is physical and emotional traces or impacts from the interaction with these locations and artifacts, illustrating how these interactions leave both tangible and intangible traces. The sense of place emerges from these interactions, reflecting the feeling or perception associated with physical locations and artifacts, and the traces they leave. Implementation outcomes such as acceptability, adoption, and sustainability are shaped by a complex interplay of these elements, whereby certain factors may amplify or diminish the influence of others, rather than simply resulting from cumulative effects.

This model has important methodological implications for studies exploring environmental contexts and implementation processes. It emphasizes the significance of physical locations, traces, and the sense of place in shaping these processes. To thoroughly investigate these aspects, it is crucial to utilize ethnographic and qualitative methods [52, 53]. These methods provide the depth and nuance needed to understand how different environmental contexts and their inherent characteristics influence implementation outcomes. Future research should focus on how the sense of place affects implementation across various contexts and changes. By leveraging these methodologies, researchers can gain insights into the complex interplay between physical locations, sense of place, and traces, as well as implementation success. Additionally, evaluating the theories developed on sense of place through a longitudinal study of the completed ED could provide deeper insights and enhance our understanding of the subject.

### Strengths and limitations

The use of multiple qualitative datasets and methods, including ethnography, in this study strengthens its validity and reliability by triangulating data from various sources, confirming findings, and enhancing the study’s credibility [53, 54]. This comprehensive approach provides a deeper understanding of how the sense of place influences implementation processes, leading to nuanced and well-supported conclusions.

Incorporating the concept of sense of place into implementation science is particularly valuable because it offers insights into how participants perceived their environments, which can have a significant impact on the acceptance and use of new changes. For implementation champions, managers, and researchers, this understanding helps tailor strategies to address both physical and emotional traces, which can be crucial for gaining acceptance and participation. Our conceptual model aims to refine the study of terms such as “context,” “setting,” and “environment” [6], thereby increasing the likelihood of successful and sustainable outcomes. In addition, our findings and model support the identification of barriers and facilitators, such as cultural resistance or environmental concerns [1, 2].

Although a sense of place can significantly influence implementation outcomes, we must recognize the limitations of generalizing findings from a single empirical study conducted in a specific location. However, this study presents theoretical concepts that offer analytical generalizability. These concepts are not confined to one context but can be applied across different settings to enhance our understanding of how the sense of place affects implementation processes. By focusing on this broader theoretical model, we can provide insights that are relevant beyond the immediate study site and contribute to a more generalized understanding of the relationship between a sense of place and implementation success. Although the sense of place may seem less direct or tangible than factors such as training or technical support, it remains a crucial element that must be balanced with practical considerations including process efficiency and adherence to new changes. Furthermore, the variability of sense of place among participants and contexts presents challenges in standardizing its impact on implementation outcomes. Effective and adaptable implementation strategies must integrate a sense of place with other practical factors while considering the generalizability of findings across different settings.

## Conclusions

The study highlights the importance of the role of a sense of place during the pre-implementation phase of changes, such as new physical layouts in an ED. Factors such as comfort, spatial organization, familiarity, time, and involvement significantly shape how participants develop their sense of place and engage with new spaces and locations. Recognizing these factors is essential for designing effective implementation processes that cater to both the physical and emotional needs of participants. Our conceptual model offers a structured framework for understanding the relationships between these theoretical concepts and factors, making it easier to identify how the sense of place affects implementation outcomes such as acceptability, adoption, and sustainability.

## Data Availability

No datasets were generated or analysed during the current study.

## References

[1] Michie S. Making psychological theory useful for implementing evidence based practice: a consensus approach. Qual Saf Health Care. 2005;14(1):26–33. 15692000 10.1136/qshc.2004.011155 PMC1743963

[2] Cane J, O’Connor D, Michie S. Others. Validation of the theoretical domains framework for use in behaviour change and implementation research. Implement Sci. 2012;7(1):37. 22530986 10.1186/1748-5908-7-37 PMC3483008

[3] Damschroder LJ, Reardon CM, Widerquist MAO, Lowery J. The updated Consolidated Framework for Implementation Research based on user feedback. Implement Sci. 2022;17(1):75. 36309746 10.1186/s13012-022-01245-0 PMC9617234

[4] Squires JE, Graham ID, Santos WJ, Hutchinson AM, The ICON, Team, Backman C, et al. The implementation in Context (ICON) Framework: a meta-framework of context domains, attributes and features in healthcare. Health Res Policy Sys. 2023;21(1):81. 10.1186/s12961-023-01028-z PMC10408185 37550737

[5] Gale NK, Shapiro J, McLeod HST, Redwood S, Hewison A. Patients-people-place: developing a framework for researching organizational culture during health service redesign and change. Implement Sci. 2014;9(1):106. 25166755 10.1186/s13012-014-0106-z PMC4147174

[6] Nilsen P, Bernhardsson S. Context matters in implementation science: a scoping review of determinant frameworks that describe contextual determinants for implementation outcomes. BMC Health Serv Res. 2019;19(1):189. 30909897 10.1186/s12913-019-4015-3 PMC6432749

[7] Wensing M. Implementation science in healthcare: Introduction and perspective. Zeitschrift für Evidenz, Fortbildung und Qualität im Gesundheitswesen. 2015;109(2):97–102. 26028446 10.1016/j.zefq.2015.02.014

[8] Anderson J. Understanding Cultural Geography: places and traces. New York: Routledge; 2015.

[9] Knight C, Haslam SA. Your place or mine? Organizational identification and comfort as Mediators of relationships between the Managerial Control of Workspace and employees’ satisfaction and well-being. Br J Manage. 2010;21(3):717–35.

[10] Mroczek J, Mikitarian G, Vieira EK, Rotarius T. Hospital Design and Staff perceptions: an exploratory analysis. Health Care Manag. 2005;24(3):233–44. 10.1097/00126450-200507000-00008 16131934

[11] Phiri M, Chen B. Sustainability and Evidence-Based Design in the Healthcare Estate. Berlin, Heidelberg: Springer Berlin Heidelberg; 2014 [cited 2020 Aug 27]. (SpringerBriefs in Applied Sciences and Technology). Available from: http://link.springer.com/10.1007/978-3-642-39203-0.

[12] Cresswell T. Place: a short introduction. Malden, MA: Blackwell Pub; 2004. p. 153. (Short introductions to geography).

[13] Massey D. Place and identity. Minneapolis:University of Minnesota Pressss; 1994.

[14] Kirk JW, Lindstroem MB, Stefánsdóttir NT, Andersen O, Powell BJ, Nilsen P, et al. Influences of specialty identity when implementing a new emergency department in Denmark: a qualitative study. BMC Health Serv Res. 2024;24(1):162. 38302985 10.1186/s12913-024-10604-0 PMC10836004

[15] Kirk JW, Stefánsdóttir NÞ, Powell BJ, Lindstroem MB, Andersen O, Tjørnhøj-Thomsen T, et al. Oilcloth sessions as an implementation strategy: a qualitative study in Denmark. BMC Med Educ. 2022;22(1):571. 35870916 10.1186/s12909-022-03635-w PMC9308909

[16] Massey D, Space. Place and gender. Minneapolis: University of Minnesota Press: Polity Press, Cambridge;; 1994.

[17] McCay, et al. Progress on the Sustainable Development Goals: the gender snapshot 2022. Erscheinungsort nicht ermittelbar: United Nations; 2022.

[18] Beebeejaun Y. Gender, urban space, and the right to everyday life. J Urban Affairs. 2017;39(3):323–34.

[19] Link A. Feminist city: claiming space in a man-made world: by Leslie Kern, New, York NY. Verso Books, 2020, 224 pp., ~$19.96 (hardcover) ISBN-10 1788739817. Soc Cult Geo. 2022;23(1):164–5.

[20] Franz C, Fratzscher M, Kritikos AS. German right-wing party AfD finds more support in rural areas with aging populations. DIW Wkly Rep. 2018;8:69–79.

[21] Nilsen P, Birken SA, editors. Handbook on implementation science. Northampton: Edward Elgar Publishing; 2020.

[22] Tuan Y. Space and place: the perspective of experience. London: In University of Minnesota Press; 1977.

[23] Seamon D, Sowers J. Place and placelessness (1976): Edward Relph. In: Hubbard P, Kitchin R, Valentine G, editors. Key texts in human geography. London: SAGE Publications; 2008. p. 43–52.

[24] Sundhedsstyrelsen. Styrket akutberedskab: planlægningsgrundlag for det regionale sundhedsvæsen [The strength of emergency preparedness: planning foundation for the regional health service]. Copenhagen: Sundhedsstyrelsen; 2007. Danish.

[25] Sundhedsstyrelsen. Anbefalinger for organisering af den akutte sundhedsindsats - Planlægningsgrundlag for de kommende [Recommendations for the organization of emergency health care - Planning basis for the next 10 years]. Copenhagen: Sundhedsstyrelsen; 2020. p. 144. Danish.

[26] Gilmour JA. Hybrid space: constituting the hospital as a home space for patients. Nurs Inq. 2006;13(1):16–22. 16494663 10.1111/j.1440-1800.2006.00276.x

[27] Marcus GE. Ethnography in/of the world system. The emergence of multi-sited ethnography. Annu Rev Anthropol. 1995;24:95–117.

[28] Hannerz U. Being there... and there... and there! Reflections on multi-site ethnography. Ethnography. 2003;4:201–16.

[29] Kirk JW, Stefansdottir NT, Andersen O, Lindstroem M, Powell BJ, Nilsen P, et al. How do oilcloth sessions work? A realist evaluation approach to exploring ripple effects in an implementation strategy. J Health Organ Manag. 2024;38(9):195–215. 38825598 10.1108/JHOM-01-2023-0022 PMC11346207

[30] World Medical Association. Declaration of Helsinki. Ethical Principles for Medical Research Involving Human Subjects. 2018. https://www.wma.net/policies-post/wma-declaration-of-helsinki-ethicalprinciples-for-medicalresearch-involving-human-subjects/. Accessed 24 Oct 2024.

[31] Tjørnhøj-Thomsen T. Samværet. Tilblivelse i tid og rum [Togetherness: Becoming in time and space]. In: Hastrup K, editor. Ind i Verden. En grundbog i antropologisk metode [Into the World. A primer in anthropological method. Copenhagen: Hans Reitzels Forlag; 2010. Danish.

[32] Clarke AE, Washburn R, Friese C, editors. Situational analysis in practice: mapping relationalities across disciplines. 2nd ed. New York: Routledge; 2022.

[33] Davidson M. Situational analysis and urban theory. Prog Hum Geog. 2023;48(2):113–30.

[34] Clarke A. Situational analysis: grounded theory after the Postmodern turn. T. housand Oaks, CA: Sage.; 2005.

[35] Coyle D. The culture code: the secrets of highly successful groups. London: Random House Business; 2019. p. 280.

[36] Allen J, Jimmieson N, Bordia P, Irmer B. Uncertainty during Organizational Change: managing perceptions through communication. J Change Manage. 2007;7(2):187–210.

[37] Kreindler SA, Dowd DA, Dana Star N, Gottschalk T. Silos and Social Identity: the Social Identity Approach as a Framework for understanding and overcoming divisions in Health Care: using the Social Identity Approach in Health Care. Milbank Q. 2012;90(2):347–74. 22709391 10.1111/j.1468-0009.2012.00666.x PMC3460209

[38] Bernhardt J, Lipson-Smith R, Davis A, White M, Zeeman H, Pitt N, et al. Why hospital design matters: a narrative review of built environments research relevant to stroke care. Int J Stroke. 2022;17(4):370–7. 34427477 10.1177/17474930211042485 PMC8969212

[39] Alansari A, Quan X. Designing High-Performance Emergency Care facilities against COVID-19. Int J Des Soc. 2022;16(2):91–113.

[40] Ulrich RS, Zimring C, Zhu X, DuBose J, Seo HB, Choi YS, et al. A Review of the Research Literature on Evidence-Based Healthcare Design. HERD. 2008;1(3):61–125. 21161908 10.1177/193758670800100306

[41] Rogers EM. Diffusion of innovations. New York; London: Free Press; Collier Macmillan; 1983.

[42] Kreitman RE. How inclusive practices in the classroom affect children’s social and emotional development. https://files.eric.ed.gov/fulltext/ED621833.pdf.

[43] Mielke J, Leppla L, Valenta S, Zullig LL, Zúñiga F, Staudacher S, et al. Unraveling implementation context: the Basel Approach for coNtextual ANAlysis (BANANA) in implementation science and its application in the SMILe project. Implement Sci Commun. 2022;3(1):102. 36183141 10.1186/s43058-022-00354-7 PMC9526967

[44] Hassanain MA. Analysis of factors influencing office workplace planning and design in corporate facilities. J Build Apprais. 2010;6(4):183–97.

[45] Kirk J, Bandholm T, Andersen O, Husted RS, Tjørnhøj-Thomsen T, Nilsen P, et al. Challenges in co-designing an intervention to increase mobility in older patients: a qualitative study. J Health Organ Manag. 2021;35(9):140–62. 33960175 10.1108/JHOM-02-2020-0049 PMC9251644

[46] Stefánsdóttir NT, Nilsen P, Lindstroem MB, Andersen O, Powell BJ, Tjørnhøj-Thomsen T, et al. Implementing a new emergency department: a qualitative study of health professionals’ change responses and perceptions. BMC Health Serv Res. 2022;22(1):447. 35382815 10.1186/s12913-022-07805-w PMC8985264

[47] Weiner BJ. A theory of organizational readiness for change. Implement Sci. 2009;4:67. 19840381 10.1186/1748-5908-4-67 PMC2770024

[48] Metz A, Boaz A, Powell BJ. A research protocol for studying participatory processes in the use of evidence in child welfare systems. Evid Policy: J Res Debate Pract. 2019;15(3):393–407.

[49] Cresswell T. Place. University of London. Elsevier.; 2009.

[50] Powell BJ, Waltz TJ, Chinman MJ, Damschroder LJ, Smith JL, Matthieu MM, et al. A refined compilation of implementation strategies: results from the Expert Recommendations for Implementing Change (ERIC) project. Implement Sci. 2015;10(1):21. 25889199 10.1186/s13012-015-0209-1 PMC4328074

[51] Waltz TJ, Powell BJ, Chinman MJ, Smith JL, Matthieu MM, Proctor EK, et al. Expert recommendations for implementing change (ERIC): protocol for a mixed methods study. Implement Sci. 2014;9(1):39. 24669765 10.1186/1748-5908-9-39 PMC3987065

[52] Proctor E, Silmere H, Raghavan R, Hovmand P, Aarons G, Bunger A, et al. Outcomes for implementation research: conceptual distinctions, Measurement challenges, and Research Agenda. Adm Policy Mental Health Mental Health Serv Res. 2011;38(2):65–76. 10.1007/s10488-010-0319-7 PMC3068522 20957426

[53] Proctor EK, Bunger AC, Lengnick-Hall R, Gerke DR, Martin JK, Phillips RJ, et al. Ten years of implementation outcomes research: a scoping review. Implement Sci. 2023;18(1):31. 37491242 10.1186/s13012-023-01286-z PMC10367273

[54] Hammersley M, Atkinson P. Ethnography. Principles in practice. 2nd ed. London and New York: Routledge; 1995.

